# LONG TERM OUTCOMES OF THE IN-HOSPITAL MOBILITY INTERVENTION (WALK FOR) IN A SAMPLE OF OLDER ADULTS

**DOI:** 10.1093/geroni/igz038.3219

**Published:** 2019-11-08

**Authors:** Anna Zisberg, Maayan Agmon, Efrat Shadmi, Efrat Gil, Yehudit Hait, Nurit Gur-Yash, Ksenya Shulyaev

**Affiliations:** 1 The Cheryl Spencer Department of Nursing Faculty of Social Welfare and Health Science, University of Haifa, Israel, Haifa, Isreal, Israel; 2 Department of Nursing, Haifa University, Haifa, Israel; 3 University of Haifa, Haifa, Israel; 4 Clalit Health Services, Haifa, Israel; 5 HaEmek Medical Center, Clalit Health Services, Afula, Israel; 6 Oranim, College of Educational, Israel., Oranim, Israel

## Abstract

Evaluation of in-hospital mobility programs is usually short-term. To examine the sustainability of Walk-FOR (Walk for Outcome and Recovery), an in-hospital mobility program in internal-medicine older (70+) patients, we conducted a quasi-experimental pre-post four-group comparative study. Walk-FOR incorporated policies encouraging patients to walk more than 900 steps/day and addressed conditions limiting patients’ in-hospital mobility. Self-reported mobility was assessed in intervention (N=159), control (N=154) and two-year follow-up groups: previous-intervention (N=75) and non-intervention (N=95) units. Two-years post-implementation, in previous-intervention units 82.7% of patients reported walking at least twice a day outside their room, similarly to the within-implementation intervention phase (81.2%, p=ns) and significantly more than in the control group (57.2%, p<.0001). No differences in walking were found between intervention and non-intervention units (84.2%, p=ns) two-years post-implementation. Multivariate analysis compering 4 study groups applying logistic regression with covariance of age, sex, walking and function ability at admission, comorbidities and length of stay demonstrated similar results. Patients from intervention units two years after it implementation had a higher odds of walking at least twice a day outside their room (OR=3.82, 95% CI 1.636-8.899, p=0.002) then patients from the same units before intervention. Logistic regression didn’t show significant differences between probability of walking at least twice a day outside their room in the group evaluated immediately after intervention implementation and two-years letter. Also there were no significant difference between not-intervention and intervention units two-years post-intervention. Walk-FOR is a sustainable practice and tends to spread to additional hospital-units probably due to hospital leadership and organizational commitment.

